# P-111. Safety, Pharmacokinetics, Pharmacodynamics and Immunogenicity of Siltartoxatug, A First-in-Class Anti-Tetanus Monoclonal Antibody: Results of Two Phase I Trials in Australian and Chinese Healthy Adults

**DOI:** 10.1093/ofid/ofaf695.339

**Published:** 2026-01-11

**Authors:** Xinyu Liu, Xiaohu Kuang, Charlotte Lemech, Zhigang Liu, Jiyuan Ding, Xia Zhou, Xiaoyi Liu, Yueming Wang, Huaxin Liao, Wanmei wang

**Affiliations:** Zhuhai Trinomab Pharmaceutical Co., Ltd., Zhuhai, Guangdong, China; Zhuhai Trinomab Pharmaceutical Co., Ltd., Zhuhai, Guangdong, China; Scientia Clinical Research Ltd, Sydney, New South Wales, Australia; The Fifth Affiliated Hospital, Sun Yat-sen University, Zhuhai, Guangdong, China; Zhuhai Trinomab Pharmaceutical Co., Ltd., Zhuhai, Guangdong, China; Zhuhai Trinomab Pharmaceutical Co., Ltd., Zhuhai, Guangdong, China; Zhuhai Trinomab Pharmaceutical Co., Ltd., Zhuhai, Guangdong, China; Zhuhai Trinomab Pharmaceutical Co., Ltd., Zhuhai, Guangdong, China; Zhuhai Trinomab Pharmaceutical Co., Ltd., Zhuhai, Guangdong, China; Zhuhai Trinomab Pharmaceutical Co., Ltd., Zhuhai, Guangdong, China

## Abstract

**Background:**

Passive immunization is recommended for tetanus prophylaxis after tetanus-prone wounds in individuals with incomplete or uncertain vaccination history. Although human tetanus immunoglobulin is standard, global supply shortages have constrained its availability. Equine tetanus antitoxin, while available in some regions, carries substantial risks of hypersensitivity reactions and has been withdrawn from developed countries. Siltartoxatug, a first-in-class monoclonal antibody targeting tetanus toxin, was developed to address these limitations as a novel passive immunizing agent. We present Phase I results from two clinical trials conducted in healthy adults in Australia (N=32) and China (N=28).

**Figure:**
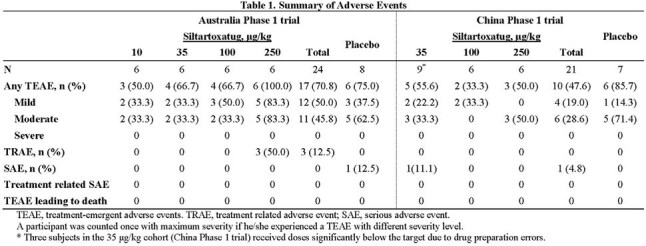

**Figure:**
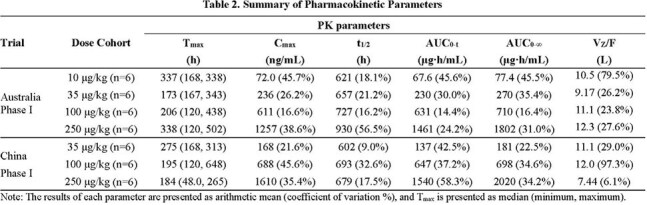

**Methods:**

Both were randomized, double-blind, placebo-controlled, dose-escalation trials, with dosing ranges of 10–250 μg/kg in Australian trial and 35–250 μg/kg in Chinese trial. Participants in each cohort were randomized 3:1 to receive a single intramuscular dose of siltartoxatug or placebo, followed by 105-day monitoring for safety, pharmacokinetics, pharmacodynamic, and immunogenicity.

**Figure:**
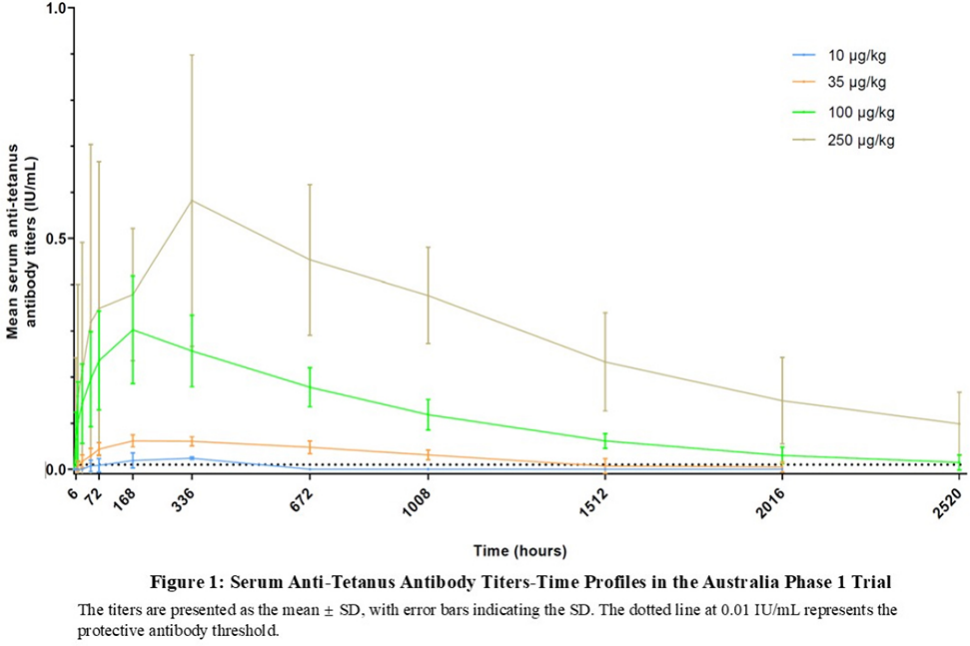

**Figure:**
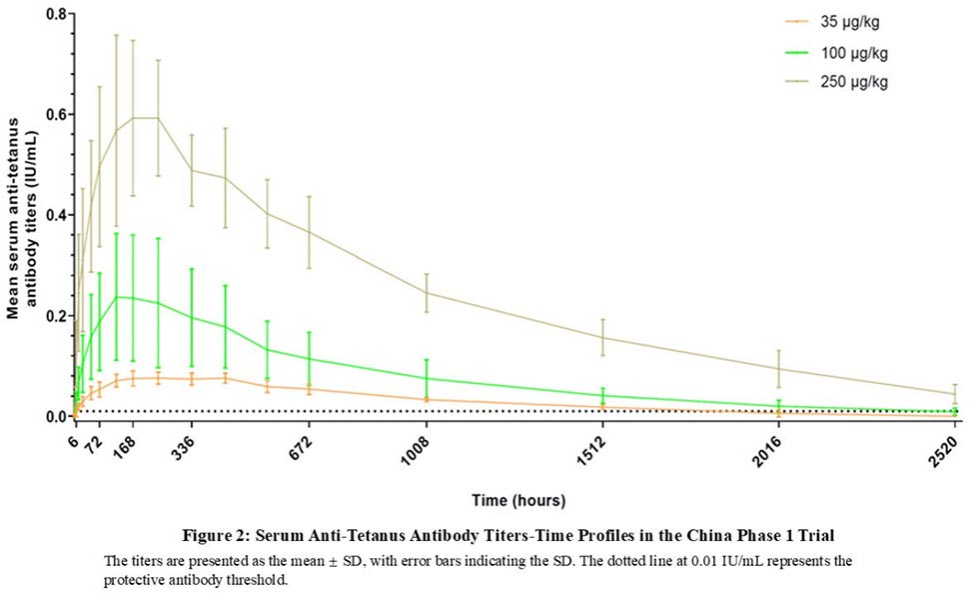

**Results:**

The incidence of treatment-emergent adverse events (TEAEs) were comparable between the siltartoxatug and placebo group in both Trials (Table 1). All TEAEs were Grade 1-2 in intensity. No treatment-related SAE was reported.

C_max_ and AUC increased dose-proportionally. The median T_max_ was 173-338 hours; and the mean t½ was 602-930 hours (Table 2). The median time to achieve anti-tetanus antibody titers ≥0.01 IU/mL (protective threshold) was < 10 hours in both the100 and 250 μg/kg dose cohorts. At 105 days post-dosing, more than 66% participants in the 100 μg/kg cohort, and 100% in the 250 μg/kg cohort maintained antibody titers ≥0.01 IU/mL. The serum antibody titer-time profiles are shown in Figure 1&2.

Anti-drug antibodies were detected in one participant with very low titers (range: 2.53-5.28), but no neutralizing antibodies were observed.

**Conclusion:**

Siltartoxatug demonstrated a favorable safety profile. Pharmacokinetics characteristics were consistent across Australian and Chinese populations, with no significant ethnic differences observed. Siltartoxatug can provide rapid and durable protective anti-tetanus antibodies, with minimal immunogenicity.

**Disclosures:**

All Authors: No reported disclosures

